# A Rare Case of Visual Hallucinations Associated With Hyponatremia

**DOI:** 10.7759/cureus.44485

**Published:** 2023-08-31

**Authors:** Vishal Sharma, Kavita Shah, Priya Reddy Mallimala, Karan Sharma

**Affiliations:** 1 Internal Medicine, Jawaharlal Nehru Medical College, Ajmer, IND; 2 Medicine, North Carolina Medical Center, Benson, USA; 3 Medicine, Kurnool Medical College, Kurnool, IND; 4 Medicine, GCS (Gujarat Cancer Society) Medical Hospital, Ahmedabad, IND

**Keywords:** gastrointestinal symptoms, hypnagogic hallucinations, electrolyte disturbances, hyponatremia, visual hallucination

## Abstract

Visual hallucinations are rare occurrences in patients presenting with hyponatremia. When they occur, the patient experiences these hallucinations with their eyes closed (vs. opened) and is insightful about the false perception. We present the case of a 64-year-old male diagnosed with hyponatremia caused by a gastrointestinal illness, which led to visual hallucinations. The patient was treated with electrolyte infusion, and the hallucinations were resolved. Detailed history-taking is significant when dealing with such cases as hallucinations to differentiate it from other causes. Hallucination caused by hyponatremia can resolve with prompt correction of sodium, and the patients can be reassured.

## Introduction

Hallucination refers to the false perception of sensory experiences. They can occur when falling asleep or waking up (like in normal cases) but also in serious conditions like dementia/schizophrenia [1]. Visual hallucination means a false perception of seeing things without any external stimulus. It can be unformed (shape/colors) or complex (figure/face). When hallucinations are substance-induced or due to psychosis, the patient is usually unaware of the reality of the perception, whereas in other causes of hallucination, the insight into reality is retained [2].

One other important feature to differentiate is closed- vs. open-eyed hallucinations. Open-eyed hallucinations are seen in psychosis, consumption of hallucinogenic substances, delirium, and hypnagogic phenomena [3], while closed-eye hallucinations, occurring when the eyes are closed, are rare and have been reported in postoperative patients who received general anesthesia or individuals with temporal lobe epilepsy [4].

Among patients with serum sodium below 120 mEq/L, 0.5% report experiencing hallucinations [5]. Recognizing these visualizations as a symptom of an electrolyte abnormality is crucial, as correcting the imbalance can resolve the hallucinations.

## Case presentation

A 64-year-old man presented with the complaint of altered sensorium and visualization for three days. Upon taking a history, it was found that he had gone on vacation with his family, where he suffered from multiple episodes of stomach ache, diarrhea, and fever. The symptoms have been progressively worsening for four days. Three days ago, he suffered from a tonic-clonic seizure. The seizure affected all four limbs. His family members noticed upward movement of eyeballs and teeth clenching. The seizure lasted for about 5 minutes. The patient was taken to a tertiary care hospital immediately. He was diagnosed with hyponatremia (Na = 93 mEq/L) and was treated for the same. Upon not getting significant improvement, the patient was transferred to our hospital.

The patient had a history of hypertension and ischemic heart disease for three years, for which he underwent percutaneous transluminal coronary angioplasty one and a half years ago.

The patient also mentioned that he has started visualizing strange people in masks approaching him when he closes his eyes. These visualizations occur when he tries to go to sleep and closes his eyes. Once he opens his eyes, the visualizations resolve. He first noticed these symptoms two nights ago while trying to go to sleep.

The patient had no history of hallucinations, psychiatric disorders, dementia, and head trauma. Although he realized his visualizations were unreal, he said they seemed real when his eyes closed.

Neurological examination revealed normal motor function, balance, and sensation. Montreal's cognitive assessment score was 28/30 (lacking 1 point in attention and 1 in delayed recall). The ophthalmologic examination was unremarkable. Laboratory reports are shown in Table 1.

**Table 1:** Laboratory results upon admission

	Patient Value	Reference Range
Sodium	118.00 mEq/L	136-145 mEq/L
Potassium	3.2 mEq/L	3.5-5.2 mEq/L
Chloride	97.70 mEq/L	98-107 mEq/L
WBC	8500/mcL	4500-11,000/mcL

Urine drug screening showed no signs of hallucinogenic substances. The urine drug test was done for cannabinoids, phencyclidine, lysergic acid diethylamide (LSD), and psilocybin.

The patient was started on electrolyte supplementation with normal saline aiming at an 8 mEq/L increase daily. A gradual decrease in the frequency of visualization was observed, and the complete resolution was achieved by the fourth day of admission. Serum sodium was 136 mEq/L on the day visualizations were resolved. The patient was discharged the next day.

## Discussion

When dealing with patients with hallucinations, it is important to remember that manifestations of underlying neurologic or ophthalmologic pathology often give rise to visual hallucinations. Thus, gathering a comprehensive medical history is crucial, paying close attention to the nature of the hallucinations, whether they are simple or complex, the specific content involved, any distortion present, any associated triggers, and the patient's insight into their reality. Examining these details allows for a more focused exploration of the potential causes of hallucinations [5].

The mechanism behind hallucinations in the case of hyponatremia can be speculated for several reasons; in general, the causes of visual hallucinations can be categorized into three groups: brain anatomy, brain chemistry, and the emergence of unconscious content in conscious perception [6]. Electrolyte abnormalities can affect both brain anatomy and brain chemistry. Hyponatremia can decrease plasma osmolality, leading to water movement into the brain in response to the osmotic gradients, thus giving rise to cerebral edema [7]. Furthermore, alterations in membrane potential can heighten the excitability of neurons in the visual cortex. One prevailing theory posits that hallucinations arise because there is a mismatch between inhibitory and excitatory forces on the brain [8].

While dealing with hallucinations in patients with electrolyte imbalance, it is important to carefully interview them to determine if they occur when their eyes are open or closed. It is also relevant to determine if the patient has an insight into the false perception.

Our patient's hallucinations were similar to hypnagogic hallucinations (commonly seen when people are falling asleep), which occur when a person transitions from wakefulness to sleep. Hypnagogic hallucinations also happen when the eyes are closed, and the patient has an insight into false perception [9]. Hypnagogic hallucinations are caused by increased thalamus activation and spontaneous discharges resulting from cortical differentiation [10]. Peck et al. suggested that hyponatremia may increase excitatory influences in the brain, leading to hallucinations [5].

This case presents a previously unreported symptom of hyponatremia, a highly prevalent issue among hospitalized patients. The correction of serum sodium levels proved to be the only necessary treatment for alleviating the hallucinations in this patient. Further investigations and interventions can be avoided unless symptoms persist or other potential causes are evident.

## Conclusions

Hallucinations associated with hyponatremia are visual and only occur with closed eyes. It should be differentiated from other causes of hallucinations by interviewing the patient, focusing on the timing of the hallucination (eyes closed vs. opened), and determining if the patient has an insight into their false perception. Hallucinations associated with hyponatremia are speculated to be caused by brain edema and an imbalance between excitatory and inhibitory influences in the brain. Correction of hyponatremia promptly leads to the resolution of symptoms.
